# Distinct gut microbiome features characterize *Fasciola hepatica* infection and predict triclabendazole treatment outcomes in Peruvian patients

**DOI:** 10.3389/fcimb.2025.1555171

**Published:** 2025-03-10

**Authors:** Giljae Lee, Bruce A. Rosa, Martha V. Fernandez-Baca, John Martin, Rodrigo A. Ore, Pedro Ortiz, Miguel M. Cabada, Makedonka Mitreva

**Affiliations:** ^1^ Division of Infectious Diseases, Department of Medicine, Washington University School of Medicine, St. Louis, MO, United States; ^2^ Division of Infectious Diseases, Department of Internal Medicine, The University of Texas Medical Branch, Galveston, TX, United States; ^3^ Sede Cusco, Instituto de Medicina Tropical “Alexander von Humboldt”, Universidad Peruana Cayetano Heredia, Cusco, Peru; ^4^ Faculty of Veterinary Science, Universidad Nacional de Cajamarca, Cajamarca, Peru; ^5^ Department of Genetics, Washington University School of Medicine, St. Louis, MO, United States; ^6^ McDonnell Genome Institute, Washington University in St. Louis, St. Louis, MO, United States

**Keywords:** liver fluke, *Fasciola hepatica*, triclabendazole, treatment response, intestinal microbiome, metagenomic shotgun sequencing, longitudinal study

## Abstract

**Background:**

*Fasciola hepatica*, a globally distributed helminth, causes fasciolosis, a disease with significant health and economic impacts. Variability in triclabendazole (TCBZ) efficacy and emerging resistance are remaining challenges. Evidence suggests that the gut microbiome influences host-helminth interactions and is associated with anthelmintic effects, but its association with human *F. hepatica* infection and TCBZ efficacy is not well understood.

**Methods:**

In this study, we investigated the relationship between *Fasciola hepatica* infection and the gut microbiome through metagenomic shotgun sequencing of 30 infected and 60 age- and sex-matched uninfected individuals from Peru. Additionally, we performed a longitudinal analysis to evaluate microbiome dynamics in relation to TCBZ treatment response.

**Results and discussion:**

Infection was associated with specific microbial taxonomic and functional features, including higher abundance of *Negativibacillus* sp900547015, *Blautia* A sp000285855, and *Prevotella* sp002299635 species, and enrichment of microbial pathways linked to survival under stress and depletion of pathways for microbial growth. Unexpectedly, we identified that responders to TCBZ treatment (who cleared infection) harbored many microbiome features significantly different relative to non-responders, both before and after treatment. Specifically, the microbiomes of responders had a higher abundance Firmicutes A and *Bacteroides* species as well as phospholipid synthesis and glucuronidation pathways, while non-responders had higher abundance of Actinobacteria species including several from the *Parolsenella* and *Bifidobacterium* genera, and *Bifidobacterium* shunt and amino acid biosynthesis pathways.

**Conclusions:**

Our findings underscore the impact of helminth infection on gut microbiome and suggest a potential role of gut microbiota in modulating TCBZ efficacy, offering novel insights into *F. hepatica*-microbiome interactions and paving the way for microbiome-informed treatment approaches.

## 1 Introduction

The liver fluke *Fasciola hepatica* is a globally distributed trematode that infects both humans and livestock (Fürst et al., 2012; Lan et al., 2024). During its life cycle in the mammalian host, *F. hepatica* undergoes a tissue-invasive phase when juveniles migrate from the intestine to the liver parenchyma and a biliary phase when adults establish a chronic infection in the biliary tree and gallbladder. Tissue damage is caused by secreted virulence factors in the excretory-secretory products (e.g., cathepsins) during intestinal penetration, liver migration, biliary tree invasion, and attachment to feed from the biliary mucosa to support egg production and release (Robinson et al., 2009; Lalor et al., 2021). These have local and systemic effects on the immune response and microbiome that may explain the clinical manifestations in the human and animal hosts (Kamel et al., 2015; Chng et al., 2016; Saltykova et al., 2016; Pawlowska et al., 2023). Infection with *Fasciola* species, known as fasciolosis, imposes significant health and economic burdens worldwide (Fürst et al., 2012; Mehmood et al., 2017). Approximately 50 million people are infected and the economic impact on the global farming industry is estimated at several billion US dollars every year (Spithill et al., 1999; Nyindo and Lukambagire, 2015).

Triclabendazole (TCBZ) is an orally-administered anthelminthic drug used to treat livestock fasciolosis and is the only drug approved for human fasciolosis (Fairweather and Boray, 1999; Saltykova et al., 2016; Gandhi et al., 2019). The precise mode of action for TCBZ against *F. hepatica* is not fully understood, but it probably involves disruption of the parasite’s tubulin polymerization, affecting essential cellular processes (Fennell et al., 2008). TCBZ undergoes metabolic transformation in the liver through sulfoxidation, producing two major metabolites-TCBZ sulphoxide and TCBZ sulphone, with the former being the most active metabolite of the drug (Virkel et al., 2006). A large body of research has reported TCBZ resistance and individual variability in drug response (Kelley et al., 2016; Morales et al., 2021; Terashima et al., 2021). Altered uptake/efflux mechanisms, metabolism into more inactivate forms, and environmental influences contribute to this variability (Robinson et al., 2004; Savage et al., 2013; Fernandez-Baca et al., 2022). TCBZ sulphoxide and TCBZ sulphone are excreted in the bile and interactions with the gut microbiome affect the equilibrium between them (Mestorino et al., 2008; Fairweather, 2009). The ruminal microbiome has been shown to transform TCBZ metabolites back to the parent compound through sulphoreduction, indicating that the gut microbiome may influence TCBZ pharmacokinetics (Virkel et al., 2006).

Helminths and bacteria have coexisted within mammalian intestine, sharing an ecological niche. There has been growing interest and studies on the effects of helminth infections on the human gut microbiome (Rosa et al., 2018; Tee et al., 2022; Silva-Caso et al., 2024). Liver flukes, including *F. hepatica*, inhabit the bile duct after penetrating the duodenum. The interaction between the bile duct microbiome and liver flukes has been studied due to their direct contact, revealing that liver fluke infection affects host microbiome, including both bile and intestinal microbiome (Plieskatt et al., 2013; Pakharukova et al., 2023). The liver and gut have bidirectional interactions via the portal vein, facilitating the translocation of gut-derived and gut microbiome-derived products, and regulating the bile acid pool. However, inconsistent results across studies from different geographical regions and infections from different helminth species make it challenging to advance our understanding of helminth-microbiome interactions. Some studies have reported microbiome alterations following anthelmintic treatment over time and suggest drug response-associated microbial features (Rosa et al., 2018; Tee et al., 2022). Notably, only one study has reported on the human gut microbiome of *F. hepatica*-infected subjects in Peru using a PCR-based approach to measure 13 bacterial genera, marking an important starting point for this research (Silva-Caso et al., 2024).

The aim of this study is to investigate the associations between the gut microbiome, *F. hepatica* infection, and anthelmintic treatment response in 114 Peruvian stool samples by leveraging metagenomic shotgun sequencing data and detailed environmental information. This metagenomic dataset enables us to capture microbial taxonomic features with high resolution, including alterations in metabolic pathways linked to infection or drug response. Our comparative analysis of Peruvian subjects with *F. hepatica* infection provides novel insights into helminth-microbiome interactions and suggests the potential role of the microbiome in modulating differential responses to anthelmintic treatment.

## 2 Materials and methods

### 2.1 Sample collection

Stool specimens of subjects with and without fascioliasis collected at baseline and after treatment were retrieved from a biorepository created for the NIAID-supported Peru Tropical Medicine Research Center grant for *Fasciola hepatica* (U01AI168622; https://tmrc-network.org/research-centers/peru). Samples were collected from rural communities of the Paucartambo province of the Cusco Region in Peru, with all participants enrolled directly from their communities without any clinic contact. Children 3 to 18 years and adults of both sexes were enrolled in a cross-sectional study of factors associated with fascioliasis transmission. After informed consent (and, if necessary, informed assent), we collected demographic, socioeconomic, and anthropometric information from subjects. Up to three stool samples and one blood sample were collected from each subject for fascioliasis diagnosis. Specimens were immediately placed in a 4-8°C thermal box and transported to the laboratory within 6 hours. Three rapid sedimentation and three Kato Katz tests were performed per stool sample (Lopez et al., 2016). The blood sample was processed to obtain serum and used for Fas2 ELISA serology for *F. hepatica* antibodies (Bionoma SRL, Lima, Peru). Aliquots of stool and serum specimens were immediately stored frozen at -80°C for subsequent use. Subjects with chronic fascioliasis were enrolled in a treatment outcomes cohort study. The main outcome of this study was parasitological cure (treatment response) as defined below. Subjects with chronic fascioliasis received directly-observed treatment in their communities with two doses of TCBZ at 10 mg/kg, separated by 24 hours and after a fatty meal following Peruvian Government treatment recommendations. Treatment response follow-up was performed 4 weeks after TCBZ administration. Subjects were asked to provide up to three stool samples for the evaluation of treatment response and each was tested using three rapid sedimentation and three Kato Katz tests.

### 2.2 Study design

For the present study, we selected consecutive cases diagnosed with chronic *Fasciola* infection and controls without it from 6 communities of the Huancarani district at an elevation of 3,850 meters. A case was defined as a subject older than 3 years regardless of sex diagnosed with chronic fascioliasis using stool microscopy. Two controls matched by sex, age (± 2 years), and, when possible, by community were selected from the same population. A control was defined as a subject from the same population without chronic fascioliasis determined by negative stool microscopy and negative Fas2 Fasciola antibody serology (Espinoza et al., 2007). Cases and controls were enrolled within a 4-week period in each community. Household geographic coordinates were used to geolocate cases and controls. The ‘get googlemap’ function from the ggmap v4.0.0 package (Kahle and Wickham, 2013) was used to generate maps of Peru depicting the location where cases and controls were enrolled.

### 2.3 Diagnosis of chronic *Fasciola hepatica* infection

Chronic infection was defined by the presence of *Fasciola* eggs in at least one stool sample identified by rapid sedimentation or Kato Katz microscopy. The uninfected controls were selected among subjects with negative stool microscopy for *Fasciola* eggs determined by at least three rapid sedimentation and three Kato Katz tests and a negative *F. hepatica* Fas2 ELISA test performed following the manufacturer’s instructions. Responders to triclabendazole (parasitological cure) were defined as cases with chronic fascioliasis with negative stool microscopy in at least three rapid sedimentation and three Kato Katz tests performed after treatment. Non-responders to TCBZ (treatment failure) were defined as cases with chronic fascioliasis with positive microscopy for *Fasciola* eggs in at least one rapid sedimentation or Kato Katz test performed after treatment. The Fas2 ELISA was not used to define response to treatment as serology can continue to be positive for several months after parasitological cure.

### 2.4 Sample selection, DNA extraction, and shipment

Banked stool specimens from cases collected at baseline and after the first triclabendazole treatment were retrieved for sequencing. Similarly, we retrieved banked baseline stool specimens from controls for sequencing. Microbial DNA extraction from stool specimens was performed using a commercially available stool DNA extraction kit (E.Z.N.A^®^ Stool DNA kit, Omega Bio-Tek Inc., Norcross, GA, USA) following the kit’s insert instructions with the following modifications. Frozen stool (200 mg) was placed in a 2 mL tube with 1.2 mL of lysis buffer, vortexed, and subjected to three cycles of freezing (−80°C for 10 minutes) and heating (90°C for 20 minutes). After this lysis step, the resulting solution was used for DNA extraction according to the manufacturer instructions. The DNA’s concentration and purity were evaluated using a NanoDrop 2000 UV–vis spectrophotometer (Thermo Scientific, Wilmington, NC, USA). After quality control, eluted DNA was mixed in GenTegra-DNA dry bulk tubes (GenTegra LLC, Pleasanton, CA, USA) following the manufacturer instructions for preservation and shipment to Washington University in St. Louis at room temperature. On arrival, dry DNA pellets were resuspended in DNase free water before a second quality control step and sequencing.

### 2.5 Metagenomic shotgun sequencing

Microbial DNA extracted from human stool samples was used for sequencing. Sequencing was performed by the Genome Technology Access Center at McDonnell Genome Institute (GTAC@MGI) at Washington University in St. Louis School of Medicine, USA. Genomic DNA (gDNA) was quantified using the Qubit Fluorometer. In brief, 250–500 ng of gDNA was fragmented using the Covaris LE220 (Covaris, Woburn, MA, USA) to achieve a mean fragment size of approximately 375 bp. Fragmented DNA was size-selected with a 0.8 ratio of Ampure XP beads (Beckman Coulter, Brea, CA, USA) to remove fragments smaller than 300 bp. Libraries were prepared using the KAPA HyperPrep Kit (Roche, Basel, Switzerland, Cat. #7962363001) and quantified with the KAPA Library Quantification Kit (Roche, Basel, Switzerland). The pooled libraries were sequenced on the NovaSeq X platform (Illumina, San Diego, CA, USA) with 150 bp paired-end reads (Modi et al., 2021).

### 2.6 Bioinformatic analysis

#### 2.6.1 Taxonomic & functional profiling

Raw sequencing reads underwent several quality-control steps before downstream profiling analysis. Illumina sequencing barcodes, adapter, and poor-quality reads were removed using Trimmomatic (Bolger et al., 2014) (v.0.36), and human contaminant reads were removed by mapping to the human reference genomes (GRCh38.98 (Martin et al., 2023)) using BMTagger (v.1.1.0). Clean reads were mapped against the Unified Human Gastrointestinal Genome (UHGG) database (Almeida et al., 2021) (v.2.0.1) using bowtie2 (Langmead and Salzberg, 2012) (v.2.5.1), followed by generating sequencing breadth and depth of coverage for every genome using InStrain (Olm et al., 2021) (v.1.5.3). Coverage depth values were normalized by dividing the depth of each genome by the total depth from all genomes, and these table was used for downstream analysis. For functional profiling, the HMP Unified Metabolic Analysis Network (HUMAnN (Beghini et al., 2021), v.3.8) pipeline was performed with the Chocophlan nucleotide database (v.201901_v31) and the Unifed90 (Suzek et al., 2015) protein database (v.201901b). The gene families table generated by HUMAnN pipeline was regrouped to MetaCyc (Caspi et al., 2020) reactions and converted to copies per million for normalization.

#### 2.6.2 Diversity analysis

For comparison of diversity between groups, a normalized species-level genome table was used to calculate Faith phylogenetic diversity values (Faith, 1992), utilizing the “Picante” package (Kembel et al., 2010) (v.1.8.2) within R environment (v.4.3.3). For the comparison of β-diversity between groups, Bray-Curtis dissimilarity matrices were calculated from the normalized genome table and visualized using non-metric multidimensional scaling (nMDS (Shepard, 1980)) with the “Vegan” package (v.2.6-8). The distances between two samples were calculated using the “metaMDSdist” function from the “Vegan” package (Dixon, 2003) and used as input for comparing distances within and between groups.

#### 2.6.3 Differential microbial feature analysis

To identify bacterial taxa or pathways that are discriminant between groups, two methods were used in this study. First, we employed linear discriminant analysis effect size (LEfSe (Segata et al., 2011); v.1.0) analysis with default settings, visualizing species-level features or bacterial pathways with an LDA score 2 and P-value 0.05 (Kruskal-Wallis test). To avoid spurious results, taxa present in fewer than 3 subjects per group were excluded from this analysis. Second, a supervised machine learning approach (Random Forest) was used to via the “randomForest” (v.4.7-1.1) package (Knights et al., 2011) in R to identify the discriminant features between groups. Discriminant power was visualized using the mean decrease in accuracy (MDA) score. The model was built with adjusted parameters (ntree=10,000), and the error rate was evaluated by out-of-bag (OOB) error. Both a normalized species-level table or MetaCyc CPM table were used as input files for both analyses.

### 2.7 Statistical analysis

Statistical comparisons of demographic characteristics and diversity index between groups were performed using GraphPad Prism software (v.10.2.3). First, the Shapiro-Wilk test was performed for each metadata to check whether datasets follow a normal distribution. For the numerical data with normality, two-sided Welch’s t-tests were used to test for statistical significance between two groups, and comparisons of proportions like sex and region were performed using Chi-square test. For the comparison of the beta dissimilarities between groups, a permutational multivariate analysis of variance (PERMANOVA) (Anderson, 2014) test was performed using “adonis2” function in “Vegan” package.

## 3 Results and discussion

### 3.1 Baseline characteristics of Peruvian subjects

**Figure 1:**
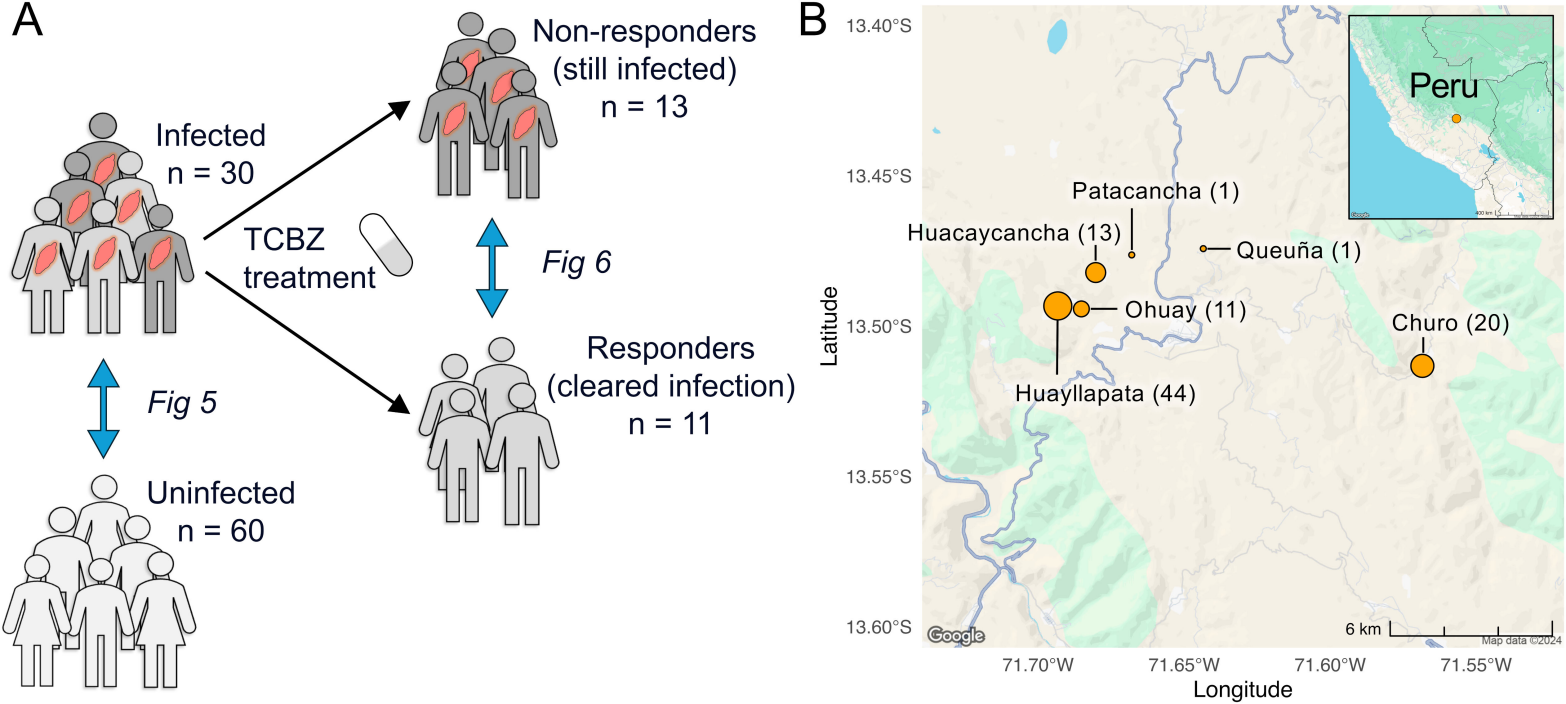
Schematic overview of study design. **(A)** This study includes 90 Peruvian subjects: 30 infected with *Fasciola hepatica* and 60 uninfected, age- and sex-matched controls. The 30 infected subjects were treated with triclabendazole (TCBZ), resulting in 13 responders, 11 non-responders, and 6 lost to follow-up. **(B)** Samples were collected from six different locations from the Cusco highlands in Peru. The number next to each region indicates the number of samples collected at that location, and the size of each circle is proportional to the sample count. Map data collected from Google Maps using the ‘ggmap’ R package (v4.0.0, in 2024).

This study includes 90 subjects, 30 of whom were chronically infected with *F. hepatica*, and 60 age- and sex-matched uninfected controls (**Figure 1A**). Samples were collected from six different locations of the Cusco highlands in Peru (**Figure 1B**). There were no statistically significant differences in subject age (Welch’s t-test, *P* = 0.938), sex (Chi-square test, *P* = 0.999), or the distribution of sample collection locations (Chi-square test, *P* = 0.623) between the infected and uninfected groups (**Table 1**).

**Table 1:** Baseline characteristics of 90 Peruvian subjects enrolled in the study.

Comparison		Infected (n=30)	Uninfected (n=60)
Sex	Female	20 (66.67%)	40 (66.67%)
	Male	10 (33.33%)	20 (33.33%)
	*P*-value (Chi-square test)		0.99
Age (years)	Min	4.1	4.5
	Max	67.55	69
	Average ± Std. dev.	20.62 ± 18.04	20.96 ± 17.83
	*P*-value (T-test)		0.93
Region	Churo	7 (23.33%)	13 (21.67%)
	Huacaycancha	3 (10%)	10 (16.67%)
	Huayllapata	16 (53.33%)	28 (46.67%)
	Ohuay	3 (10%)	8 (13.33%)
	Patacancha	0 (0%)	1 (1.67%)
	Queuña	1 (3.33%)	0 (0%)
	*P*-value (Chi-square test)		0.62

The 30 subjects with chronic *F. hepatica* infection were treated with two doses of TCBZ and treatment response was evaluated four weeks later by stool microscopy. Of these, 13 subjects had negative stool microscopy for *F. hepatica* (responders), 11 had positive stool microscopy (non-responders), and six did not provide follow-up samples. There were no significant differences in the duration of time between the initial visit and final follow-up (Welch’s t-test, *P* = 0.854) or between treatment and final visit between the two groups (*P* = 0.978, **Table 2**). *In vitro* susceptibility studies in the Cusco area using livestock isolates suggested seasonality in the prevalence of resistance to the TCBZ (Fernandez-Baca et al., 2022), but we did not identify any significant seasonal collection bias in our study (Chi-square test, *P* = 0.284).

**Table 2:** Demographic characteristics of subjects classified by the respondence on the treatment.

Comparison		Responder (n=11)	Non-responder (n=13)
Sex	Female	8 (72.73%)	7 (53.85%)
	Male	3 (27.27%)	6 (46.15%)
	*P*-value (Chi-square test)		0.34
Age (years)	Min	4.1	4.26
	Max	46.62	67.55
	Average ± Std. dev.	19.24 ± 15.87	21.4 ± 20.76
	*P*-value (T-test)		0.78
Region	Churo	4 (36.36%)	2 (15.38%)
	Huacaycancha	2 (18.18%)	1 (7.69%)
	Huayllapata	5 (45.45%)	10 (76.92%)
	Ohuay	0 (0%)	0 (0%)
	Patacancha	0 (0%)	0 (0%)
	Queuña	0 (0%)	0 (0%)
	*P*-value (Chi-square test)		0.28
Duration of time (days)	Initial to final follow-up	92.09 ± 27.3	89.38 ± 43.09
	*P*-value (T-test)		0.85
	Treatment to final follow-up	35.64 ± 14.56	35.92 ± 31.15
	*P*-value (T-test)		0.98
Month	Date of treatment		
	April	5 (45.45%)	10 (76.92%)
	July	2 (18.18%)	1 (7.69%)
	October	4 (36.36%)	2 (15.38%)
	*P*-value (Chi-square test)		0.28
	Date of follow up		
	April	5 (45.45%)	9 (69.23%)
	July	2 (18.18%)	2 (15.38%)
	October	4 (36.36%)	2 (15.38%)
	*P*-value (Chi-square test)		0.43

### 3.2 Comparison of microbial communities in relation to *F. hepatica* infection and TCBZ treatment response

To profile the human gut microbial community in association with *F. hepatica* infection, we performed metagenomic shotgun sequencing on 114 samples collected from 90 subjects, consisting of 60 samples from uninfected controls and 54 samples from 30 infected subjects collected before and after treatment, as described above. An average of 21 million paired-end reads were generated per sample, and after processing and mapping reads (see methods), 1528 unique species-level representative genomes (from the UHGG database (Almeida et al., 2021)) and 491 MetaCyc (Caspi et al., 2020) metabolic pathways were detected across all samples. Sample metadata, total read counts, NCBI SRA accessions (Katz et al., 2022) (BioProject PRJNA1194543) and normalized relative abundance values per genome and pathway are provided for all samples in **Supplementary Table S1**.

**Figure 2:**
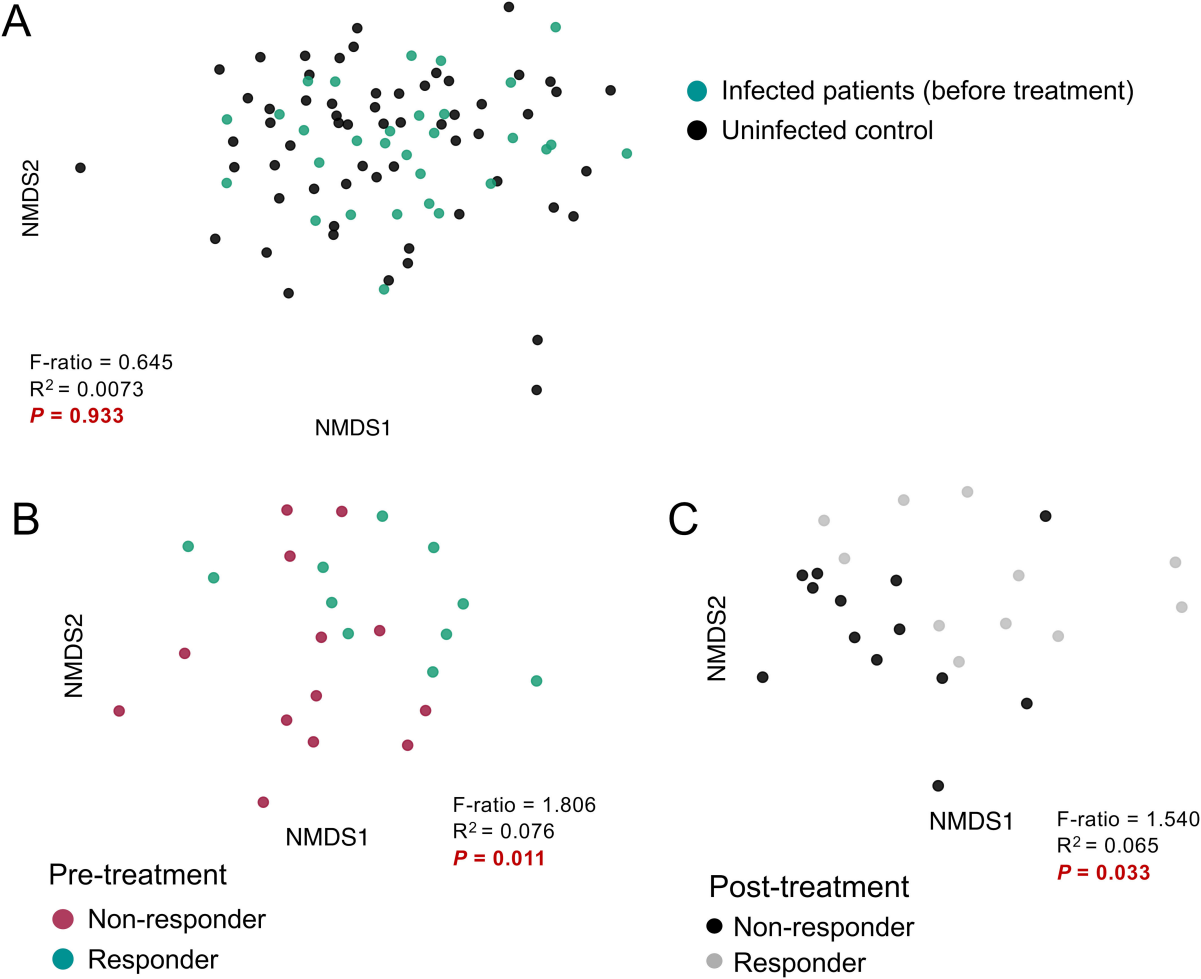
Comparisons of beta-diversity by infection status, and treatment response. Microbiome beta diversity comparisons grouped by **(A)** pre-treatment infection status, **(B)** infected pre-treatment responders and non-responders, and **(C)** post-treatment responders and non-responders. Microbiome composition was compared using non-metric multidimensional scaling (NMDS) analysis based on Bray-Curtis distances. Statistical significance between groups was calculated using the permutational multivariate analysis of variance (PERMANOVA) test.

We used the Bray-Curtis dissimilarity index to compare the similarity of microbial communities between groups and visualized the results using NMDS. Before treatment, infected subjects did not show significant separation compared to the uninfected controls (PERMANOVA test, *P* = 0.933, **Figure 2A**). However, significant differential clustering (*P* = 0.011) was observed between the baseline microbiome of responders and non-responders to the treatment (**Figure 2B**), and this difference persisted after treatment (*P* = 0.033, **Figure 2C**). These results suggest that TCBZ responders and non-responders have distinct baseline gut microbiome profiles before treatment, and that these differences persist even after treatment, suggesting an association between the gut microbiome and *F. hepatica* TCBZ resistance which is present and detectable prior to treatment.

**Figure 3:**
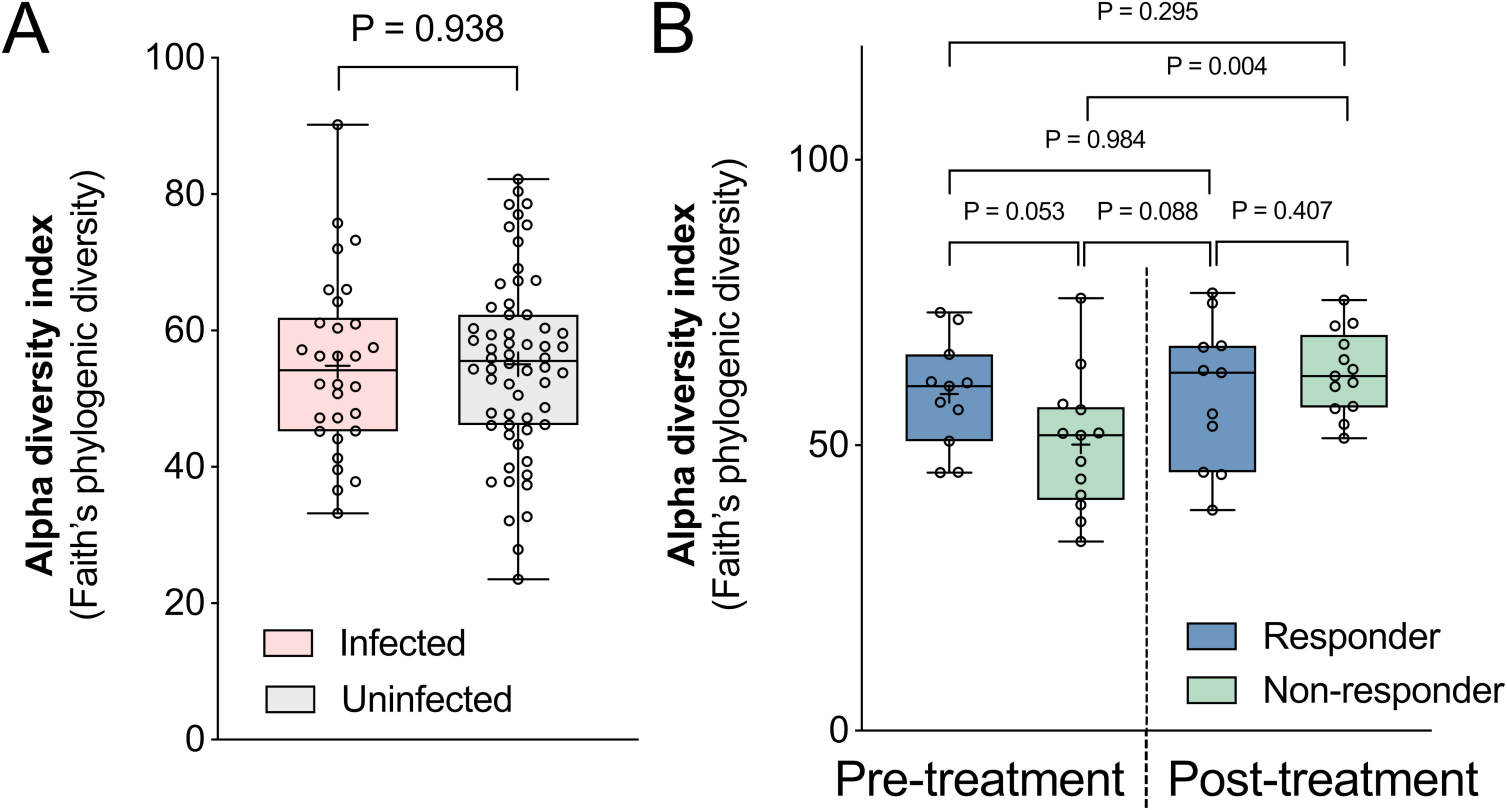
Within-sample (alpha) diversity comparisons by infection status and treatment response. Differences in alpha diversity were calculated between samples grouped by **(A)** pre-treatment infection status, and **(B)** responders and non-responders before and after TCBZ treatment. Alpha diversity index was calculated based on Faith’s phylogenic diversity index. The box plot displays the median, 25th to 75th percentiles, with whiskers extending from min to max. Statistical significance between groups was calculated using Welch’s t-tests.

We next examined differences in Faith’s phylogenetic diversity (Faith’s PD), a measure of within-sample diversity that accounts for the phylogenetic relatedness within the microbiome. No differences were observed based on infection status (Welch’s t-test, *P* = 0.938, **Figure 3A**), and although non-responders had lower pre-treatment alpha diversity compared to responders (**Figure 3B**), this difference was not statistically significant (*P* = 0.053). Unexpectedly, the within-sample diversity of non-responders significantly increased after TCBZ treatment despite not clearing the *F. hepatica* infection (*P* = 0.004), while no significant difference was observed for the responders. The time interval between post-treatment sampling did not differ between the two groups (Welch’s t-test, *P* = 0.977, **Table 2**). Recent research has revealed that the response to immunotherapy in melanoma patients is correlated with gut microbiome stability during treatment, with stable microbial functions supporting therapeutic effect (Macandog et al., 2024), which aligns with our observation and again suggests a possible important role for the microbiome in modulating TCBZ treatment success. Further supporting this hypothesis, previous research has demonstrated associations between the gut microbiome and the efficacy of anthelmintic drugs in helminth-infected subjects (Schneeberger et al., 2022; Appiah-Twum et al., 2023). While these studies primarily focused on intestinal worms, they revealed similar patterns: a specific enterotype was linked to the efficacy of albendazole-ivermectin against whipworm and hookworm in a multi-country study (Schneeberger et al., 2022), and microbial taxa were associated with infection clearance by albendazole in Ghanaian individuals with hookworm infections (Appiah-Twum et al., 2023). Together, these findings underscore the potential importance of the microbiome in modulating the efficacy of TCBZ and other anthelmintic therapies. More detailed analysis of specific microbiome taxa and pathways in each of these comparisons is provided below.

**Figure 4:**
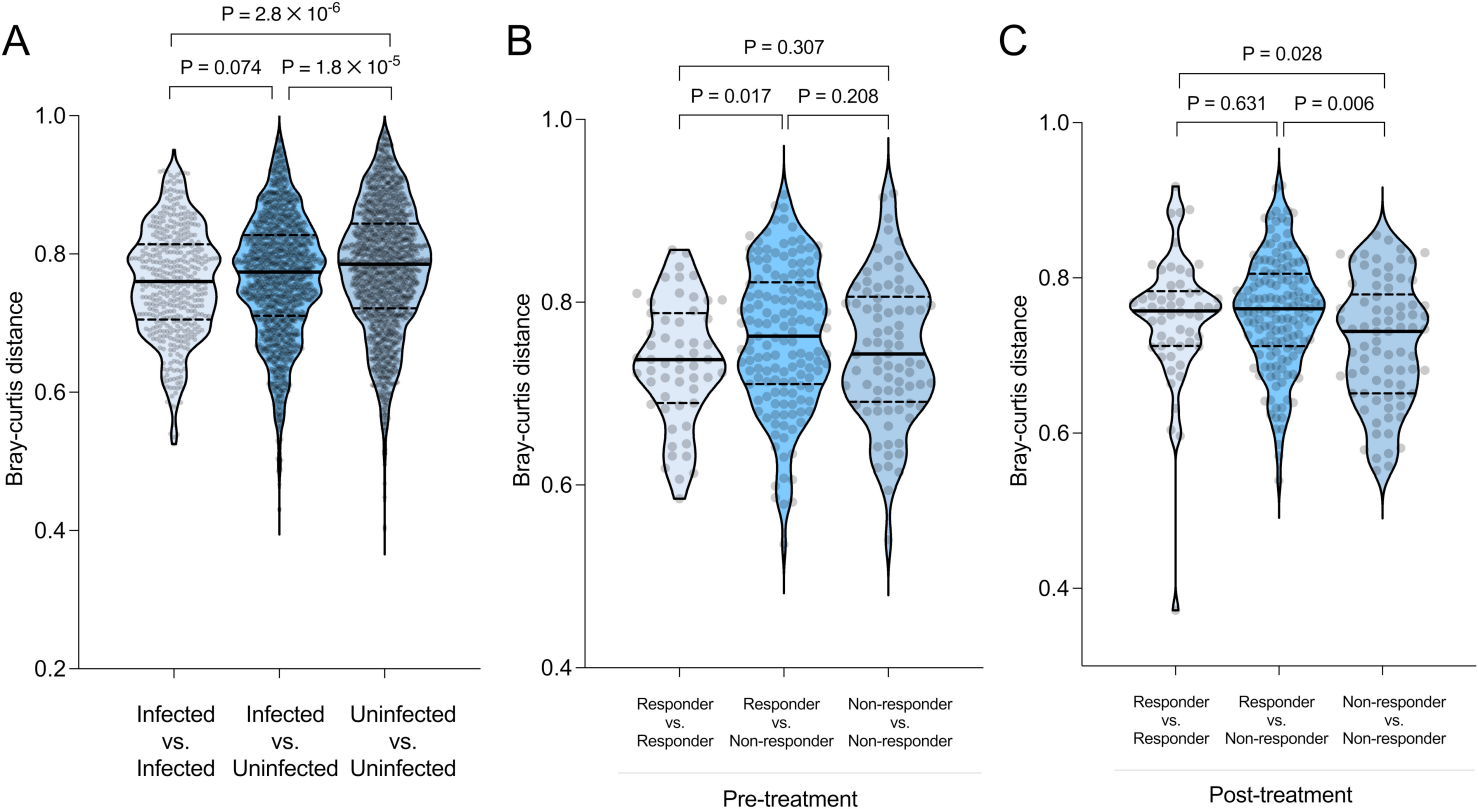
Comparison of between-sample (beta) diversity using Bray-Curtis dissimilarities within and between groups. Beta diversity within and between groups was compared using the Bray-Curtis dissimilarity matrix, grouped by **(A)** infection status and treatment response, **(B)** before treatment, and **(C)** after treatment. The violin plots display the median (dashed line), 25th and 75th percentiles (dotted lines), with whiskers extending from the minimum to maximum values. Data distribution is visualized with wider sections (indicating higher data density) or narrower sections (indicating lower density). Statistical significance between groups was determined using Welch’s t-test.

To further compare microbiome similarities within and between groups, we calculated Bray-Curtis dissimilarity values between each sample pair in the dataset, and compared the results for different groups of samples (**Figure 4**). Pre-treatment infected patients had a significantly more similar microbiome to each other compared to those of uninfected subjects (Welch’s t-test, *P* = 2.9 ×10^-6^, **Figure 4A**), suggesting that infected samples tend towards a shared microbiome profile, relative to the diversity among uninfected samples. No significant difference was observed between the infected responders and non-responders before treatment (*P* = 0.307, **Figure 4B**). However, after treatment, non-responders, who remained infected, had significantly more similar microbiomes to each other compared to responders who cleared the infection (*P* = 0.028, **Figure 4C**). Taken together, these results show that infected subjects tend to have similar microbiome profiles to each other prior to treatment, and that their microbiomes diverge after successful TCBZ administration.

### 3.3 Gut microbial species and pathways associated with *F. hepatica* infection

**Figure 5:**
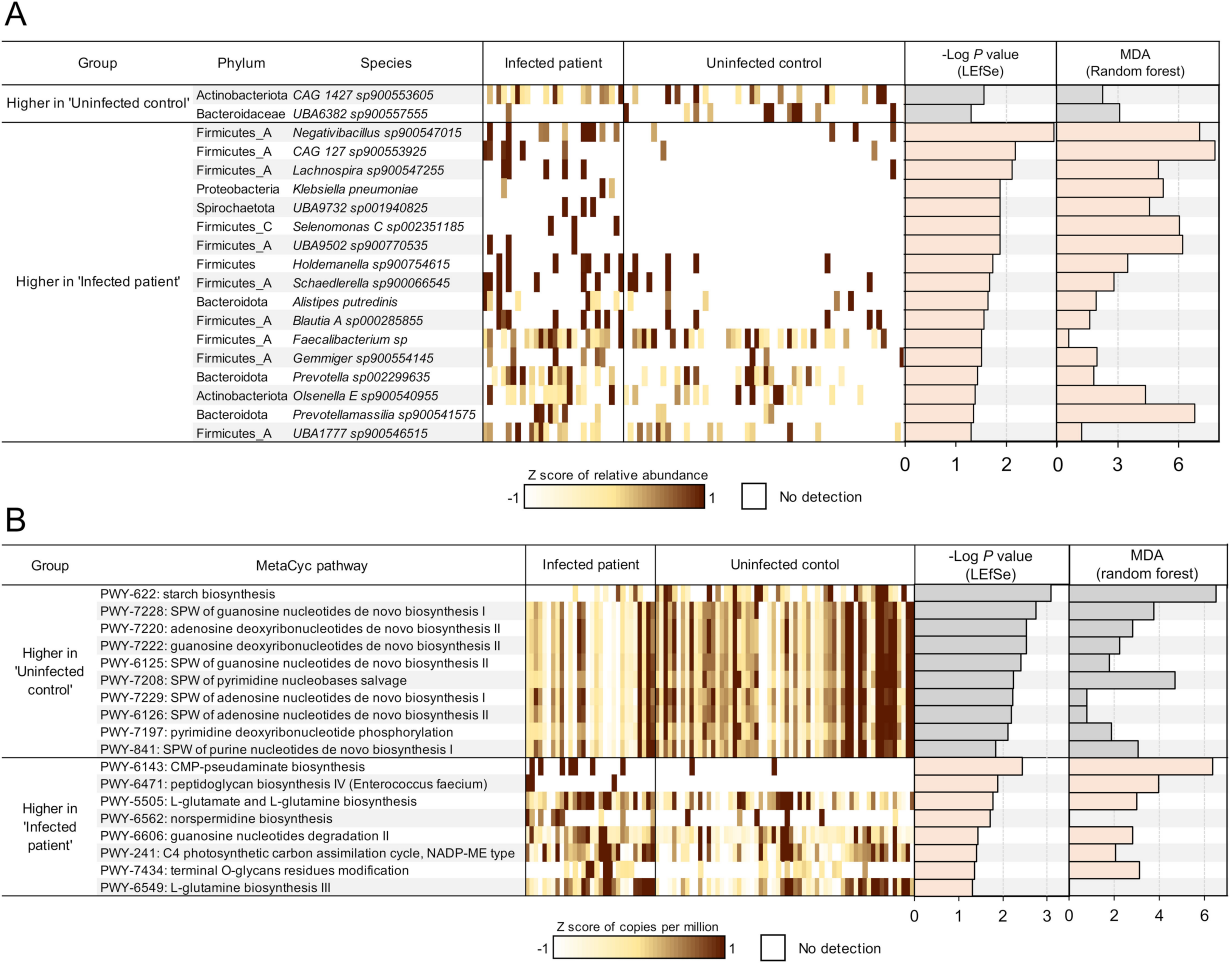
Differential microbial features by *F hepatica* infection status. **(A)** Microbial species or **(B)** metabolic pathways significantly differentially abundant between *F hepatica*-infected patients and uninfected controls at both timepoints, based on LEfSe analysis. Features with LDA score 2 and *P*-value < 0.05 are ranked by -Log of *P*-value, and normalized expression values (copies per million) were transformed to z-scores to show the abundance of each feature in each sample (1 sample per column). Additionally, the -Log of P-value from LEfSe and the mean decrease of accuracy (MDA) value from random forest analysis are presented as bar graphs. SPW, super pathway.

Next, we identified differential microbial species and bacterial metabolic pathways based on infection status. Significant differential features were identified according to LEfSe (Segata et al., 2011) analysis (LDA score 2 and Kruskal-Wallis test *P*-value 0.05), and additional validation of the association was provided by examining the ‘mean decrease of accuracy’ (MDA) scores measuring the importance of each feature in Random Forest (RF) machine learning analysis (**Figure 5**). To identify consistent microbial features associated with treatment response, we focused on features that overlapped across time points; a) significantly associated with pre-treatment infected vs uninfected samples, and b) significantly associated with post-treatment infected non-responders vs responders. The significant features associated with infection in each comparison are shown in **Figure 5A**. For the microbial species comparisons, differential expression statistics for each comparison, relative abundance values (normalized depth), and complete phylogeny data are provided for each taxonomic feature across all samples in **Supplementary Table S2**. For MetaCyc (Caspi et al., 2020) pathway comparisons, differential expression statistics and relative abundance values (copies per million, CPM) are provided in **Supplementary Table S3**.

This analysis identified 19 significantly differential species between infected and uninfected subjects, with most species showing a higher abundance in infected patients before treatment (**Figure 5A**). Species associated with infection included a species of *Blautia* (*Blautia A* sp*000285855*, *P* = 0.028), a genus which has been shown to be positively associated with soil transmitted helminth (STH) infection in patients from Indonesia and Liberia (Rosa et al., 2018) as well as a species of *Prevotella* (*Prevotella* sp*002299635*, *P* = 0.038), a genus which is among the most often cited as being associated with helminth infection (based on a review paper and meta-analysis on the topic (Kupritz et al., 2021)), and which has been associated with autoimmune disease (Scher et al., 2013). Five of 11 species significantly higher in the infected group belong to the Ruminococcaceae/Lachnospiraceae family, including *Negativibacillus* sp*900547015* (*P* = 1.2 × 10^-3^), *Gemmiger* sp*900554145* (*P* = 0.031), and *Faecalibacterium* sp (*P* = 0.031). These families were also found to be increased in a hamster model infected with *Opisthorchis viverrini*, a carcinogenic liver fluke, six weeks post-infection (Plieskatt et al., 2013). The genus *Negativibacillus* has been associated with inflammatory bowel disease (IBD), including refractory IBD (Dovrolis et al., 2020; Gryaznova et al., 2021). In an experimental mouse study, *Negativibacillus* was correlated with shortening of the intestinal tract, which is an indicator of poor gut health and impaired nutrient absorption (Wu et al., 2022). However, previous research has not linked this genus to any helminth infections. Additionally, the increased abundance of *Prevotella* has been associated with autoimmune disease (Scher et al., 2013). The bile duct microbiome has been closely associated with bile-duct related disease, with microbiome profile variations being dependent on the etiology (Tyc et al., 2020). Studies analyzing the bile duct microbiome in liver diseases, including those associated with liver fluke infection, have commonly shown an increase in Proteobacteria (Chng et al., 2016; Saltykova et al., 2016). In this study, *Klebsiella pneumoniae* levels were higher in association with *F. hepatica* infection, suggesting a close interaction between the gut and bile microbiomes (*P* = 0.013). These previous studies utilized 16S sequencing, so direct comparisons to our metagenomic shotgun sequencing samples are not possible. In contrast, *Faecalibacterium* (Martín et al., 2023), *Alistipes putredinis* (Ishikawa et al., 2024), and *Gemmiger* (Palmieri et al., 2024), all of which are higher with *F. hepatica* infection, are generally recognized as beneficial for their anti-inflammatory properties. The complex changes in these bacteria may reflect gut dysbiosis, where normal balance of the microbiome is disrupted.

When deduced microbial functional profiles between infected and non-infected subjects before treatment were compared, a total of 18 distinct metabolic pathways were differentially abundant (**Figure 5B**). Notably, 10 pathways were more abundant in uninfected subjects, including starch biosynthesis and 9 closely related pathways associated with nucleotide synthesis, energy metabolism, and cellular proliferation, suggesting that essential pathways for microbial growth were suppressed in infected environments. In contrast, pathways related to L-glutamate and L-glutamine (PWY-5505, *P* = 0.017 and PWY-6549, *P* = 0.048) were upregulated in infected subjects. L-glutamate and L-glutamine are key amino acids involved in nitrogen metabolism and energy production (Walker and van der Donk, 2016). These amino acids also facilitate biofilm synthesis (Kimura and Kobayashi, 2020), which aligns with the upregulation of the norspermidine biosynthesis (PWY-6562, *P* = 0.020), since norspermidine is involved in biofilm formation (Wotanis et al., 2017). The terminal O-glycan residues modification pathway (PWY-7434, *P* = 0.044) is typically associated with eukaryotes; however, the microbiome can exhibit glycan-modifying activities that influence host responses such as inflammation (Yamada et al., 2019). Most of the enriched microbial pathways in infected subjects were related to response to harsh environment or inflammation conditions. Overall, this suggests that the gut environment in infected patients may be stressful for gut microbiome, leading to a focus on survival rather than microbial growth and DNA replication.

### 3.4 Specific gut microbial species are associated with differential *F. hepatica* responses to TCBZ treatment, both before and after treatment

**Figure 6:**
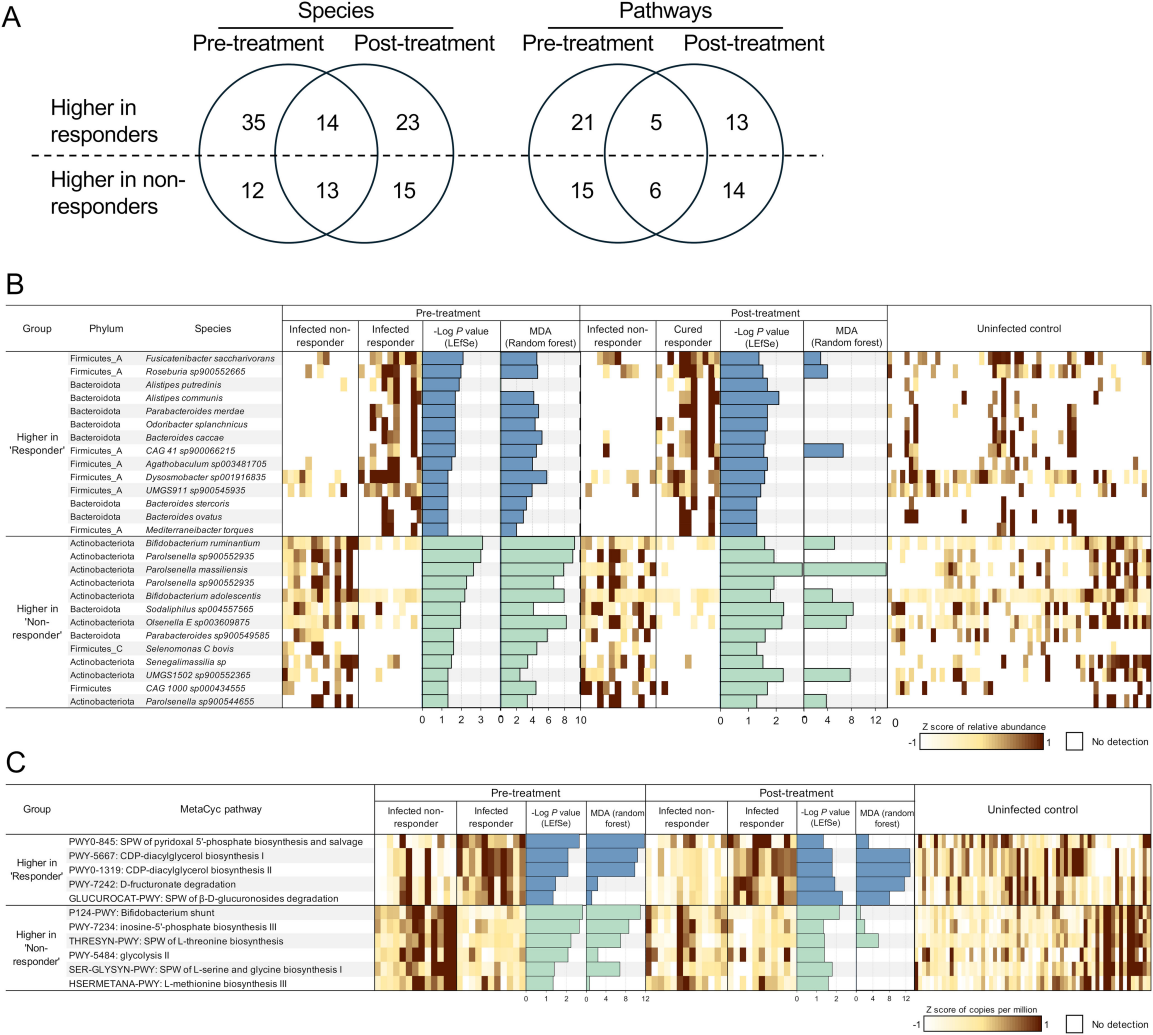
Differential microbial features by treatment response before and after treatment. **(A)** Counts of microbial species and pathways significantly associated with responder samples or non-responder samples, both before and after treatment. **(B)** Microbial species or **(C)** metabolic pathways significantly differentially abundant between responders and non-responders at both timepoints, based on LEfSe analysis. Features with LDA score 2 and *P*-value < 0.05 are ranked by -Log of *P*-value, and normalized expression values (copies per million) were transformed to z-scores to show the abundance of each feature in each sample (1 sample per column). Additionally, the -Log of P-value from LEfSe and the mean decrease of accuracy (MDA) value from random forest analysis are presented as bar graphs. SPW: super pathway.

To identify differential microbial features between non-responders and responders, we characterized microbial features that were significantly associated with each group both before and after treatment. We identified a total of 27 overlapping species-level genomes associated with either responders (13) or non-responders (14) both before and after TCBZ treatment, along with many that were significantly only pre-treatment or only post-treatment (**Figure 6A**).

Species from the phylum Firmicutes were enriched in responders, represented by 7 of the 14 species higher in both, before and after treatment. Three *Bacteroides* species (*B. caccae*, *P* = 0.049 for both before and after treatment; *B. stercoris*, *P* = 0.049 for both; and *B. ovatus, P* = 0.020, before treatment, *P* = 0.025, after treatment) were more abundant in responders. *Bacteroides* is a highly abundant and variable genus, known for its role in drug metabolism (Shu et al., 1991; Zimmermann et al., 2019). Zimmermann et al. screened the ability of human commensal bacteria to metabolize 271 oral drugs, finding that *B. thetaiotaomicron* and *B. dorei* metabolized 46 and 16 drugs, respectively (Zimmermann et al., 2019). Another study reported that major *Bacteroides* and *Clostridium* species metabolized the anthelmintic drug levamisole, enhancing its activity (Shu et al., 1991). These findings suggest that responders and non-responders have distinct microbiome niches, with a *Bacteroides*-enriched microbial environment in responders and an Actinobacteria-enriched environment in non-responders. This distinction likely results in different functional potentials of the gut microbiome, which may be associated with varying responses to drug treatment.

In non-responders (who retained *F. hepatica* infection following TCBZ treatment), 9 of the 13 species significantly more abundant than in responders belonged to the phylum Actinobacteria (**Figure 6B**). Among the 13 species significantly more abundant in the non-responders, 4 were classified within the *Parolsenella* genus. Interestingly, these species were not detected at all in the responders, except for one individual who had *P. massiliensis* after treatment at a very low relative abundance of 9.2 × 10^-4^%. In contrast, the average abundance and prevalence of *P. massiliensis* in non-responders were 0.569% and 61.54%, respectively, before treatment, and 0.277% and 76.92%, respectively, after treatment. The functions, pathogenicity, and benefits of these species remain largely unexplored. The abundance of *P. catena*, a *Parolsenella* species, has been associated with symptomatic human giardiasis in Iranians (McGregor et al., 2023), while *P. massiliensis* was reported to be negatively associated with BMI, allergies, and intestinal illness in non-industrialized Hondurans (Shridhar et al., 2024). Two *Bifidobacterium* species, *B. ruminantium* (*P* = 8.2 × 10^-4^ before treatment and *P* = 0.026 after treatment) and *B. adolescentis* (*P* = 0.006 before treatment and *P* = 0.016 after treatment), were also significantly enriched in non-responders. *B. adolescentis* is a prevalent species in the human gut, specialized in metabolizing resistant starch (Duranti et al., 2014) and producing short-chain fatty acids (Amaretti et al., 2007), which are correlated with health benefits, including anti-inflammatory effects (Schirmer et al., 2016; Leser and Baker, 2023). A previous PCR-based study also identified a significant association of *Bifidobacterium* (at the genus level) with *F. hepatica* infection in children from Peru (Silva-Caso et al., 2024), but here we identify specific species to support these findings. Another study analyzing human the bile duct tissue microbiome of cholangiocarcinoma patients revealed that Bifidobacteriaceae was enriched in *Opisthorchis viverrini*-associated cancer patients compared to non-*O. viverrini*-associated cancer patients (Chng et al., 2016), possibly suggesting translocation of enteric bacteria into bile duct.

One species of *Olsenella* (*O. E* sp*003609875, P* = 0.011 before treatment and *P* = 0.006 after treatment) was also significantly higher with non-responders and was previously associated with STH infection in patients from Indonesia and Liberia, with a significant reduction only after successful treatment (Rosa et al., 2018).

Overall, the striking differences in microbial communities’ structure and function between responders and non-responders, particularly in *Bacteroides* and Actinobacteria species, suggest that the pre-existing gut microbiome features may influence TCBZ treatment outcomes. The persistence of these differences after treatment, even as the responders clear their infections, further supports a potential mechanistic link between specific gut bacterial communities and TCBZ efficacy rather than these differences being merely a consequence of successful treatment.

### 3.5 Gut microbial pathways are also associated with TCBZ response both before and after treatment

To further investigate the bacterial functional characteristics in the gut environment of non-responders, we performed LEfSe analysis, which revealed that 11 bacterial metabolic pathways were enriched in either responders (5) or non-responders (6), consistently both before and after the TCBZ treatment (**Figure 6C**).

Pathways related to phospholipid synthesis (PWY-5667 and PWY0-1319, *P* = 0.008 before treatment and *P* = 0.016 after treatment for both; crucial for microbial cell membrane formation), nutrient processing (PWY-7242, *P* = 0.034 before treatment and *P* = 0.012 after treatment) and the breakdown of β-D-glucuronosides (GLUCUROCAT-PWY, *P* = 0.046 before treatment and *P* = 0.005 after treatment), were significantly more abundant in responders vs. non-responders both before and after treatment. Glucuronidation is one of the liver detoxification pathways, conjugating glucuronic acid to various compounds such as toxins, drugs, or xenobiotics, making them more water-soluble for excretion via urine or bile (Wells et al., 2004). However, these excreted conjugated compounds can be deconjugated by gut microbial *β*-glucuronidase (gmGUS) in the intestine, regenerating the parent compounds and reactivating through enterohepatic circulation (Gao et al., 2022). In this study, the level of gmGUS (EC 3.2.1.31) was compared between responders and non-responders. Although baseline levels of this enzyme were lower in non-responders, the difference was not significant (Mann-Whitney U test, *P* = 0.119). However, after treatment, the enzyme level was significantly lower in non-responders (*P* = 0.006; **Supplementary Figure S1**). To date, research on gmGUS enzymes has primarily focused on the context of inhibiting this enzyme to reduce gastrointestinal damage caused by reactivated drug-induced toxicity or toxic substances (Wallace et al., 2010; Zhang et al., 2022). However, TCBZ is considered relatively tolerable in humans (El-Tantawy et al., 2007; Terashima et al., 2021). Varying microbial enzymatic responses to the treatment may be linked to individual differences in TCBZ clearance. However, in this study we are not measuring the metabolism or clearance of TCBZ, so further research is needed to elucidate the causal effect of these enzymatic responses and to determine any possible differences in TCBZ inactivation or sulphoreduction in the presence of distinct microbiome profiles.

In non-responders, pathways such as the *Bifidobacterium* shunt (P124-PWY; also known as the fructose-6-phosphate pathway, a fermentation pathway used to identify *Bifidobacterium* (Caspi et al., 2020), *P* = 0.002, *P* = 0.007 before and after treatment, respectively) and amino acid biosynthesis pathways, including threonine biosynthesis (THRESYN-PWY, *P* = 0.006, *P* = 0.040 before and after treatment, respectively), serine and glycine biosynthesis (SER-GLYSYN-PWY, *P* = 0.040, *P* = 0.016 before and after treatment, respectively), and methionine biosynthesis (HSERMETANA-PWY, *P* = 0.046, *P* = 0.026 before and after treatment, respectively), were significantly more abundant compared to responders. The higher abundance of the *Bifidobacterium* shunt pathway reflects the enrichment of bifidobacterial species in non-responders (including *B. ruminantium* and *B. adolescentis*).

The contrasting pathway abundances between responders and non-responders, particularly higher drug metabolism and membrane-related processes in responders versus basic biosynthesis in non-responders, suggest that gut microbial functions may influence TCBZ efficacy, possibly through direct drug modification or altered host-drug interactions.

## 4 Conclusion

For the first time, we have analyzed the gut microbiome features (both species and pathways) associated with *Fasciola hepatica*-infected patients in Peru, using a relatively large cohort of metagenomic shotgun sequencing samples. Infected patients were distinguished from uninfected patients by specific microbial species and metabolic pathways (without community-level changes), but unexpectedly, using our cohort of samples before and after TCBZ anthelmintic treatment, we also identified a number of specific microbiome features significantly associated with non-responders who remained infected, relative to responders who cleared infection, both before and after treatment. While it was expected that microbiome features would differ between these groups after treatment, it was surprising to identify 27 genomes and 11 pathways that were consistently differential between the cohorts even before treatment, when they were both infected with *F. hepatica*. While we cannot make conclusive statements about causality without an experimental model of infection, these results strongly indicate that TCBZ treatment efficacy is either directly influenced by the gut microbiome, or that an unknown biological mechanism correlates both with the gut microbiome and with TCBZ treatment success. These findings suggest potential opportunities for microbiome-based prediction of TCBZ treatment success prior to administration, as well as potential microbiome-targeted interventions to improve TCBZ efficacy. The specific microbial species and pathways identified here provide a valuable foundation for future studies on this topic, and all data analyzed has been made easily accessible in the **Supplementary Tables** for other researchers.

Collecting human gut microbiome samples from *Fasciola* endemic areas for sequencing is difficult, and while we have collected a relatively large sample size of infected and uninfected samples both before and after treatment for a study of this kind (Walusimbi et al., 2023), future controlled studies to establish causality or prospective studies to validate TCBZ response predictions with larger cohorts will help to statistically validate the findings observed here. Research on *F. hepatica*-microbiome interactions in humans is particularly limited, largely due to the difficulty in obtaining human samples. Most studies on fascioliasis lack longitudinal follow-up of diagnosed subjects. However, we address this gap by conducting follow-up directly within the communities where we treat the subjects and utilizing a shotgun metagenomic approach, which enables high-resolution taxonomic and functional profiling of the human microbiome. Our analysis indicates associations without being able to indicate causality, but the specific findings in terms of bacterial species and metabolic pathways provide a valuable resource for future causative studies. Moreover, as our analysis focuses on a single region in Peru, further studies comparing different locations or countries with different baseline microbiomes will provide insights into microbial features associated with *F. hepatica* infection or TCBZ response that are independent of region or cohort. Additionally, collecting bile from the biliary tree for microbiome analysis using invasive techniques such as endoscopic retrograde cholangiopancreatography would offer better insights into the changes induced by *F. hepatica* infection in its immediate environment. However, the invasiveness of such procedures and their significant complications including pancreatitis, perforations, and bleeding make collection of such samples among human subjects with few symptoms in the community unjustifiable. Overall, our temporal analysis has allowed us to quantify and characterize human gut microbiome features significantly associated with *F. hepatica* infection as well as with resistance to TCBZ treatment. These results provide novel insights into helminth-microbiome interactions that may lead to future strategies for predicting TCBZ efficacy prior to treatment, and to aiding in curing and preventing *F. hepatica* infections. More broadly, this work provides a framework for understanding how the gut microbiome may influence anthelmintic drug efficacy across other helminth infections, highlighting how pre- and post-treatment sampling can reveal key microbiome associations with treatment outcomes.

## Data Availability

The datasets presented in this study can be found in online repositories. The names of the repository/repositories and accession number(s) can be found in the article/**Supplementary Material**.
