# Supplementary material for: Distinct gut microbiome features characterize Fasciola hepatica infection and predict triclabendazole treatment outcomes in Peruvian patients
Source: Front Cell Infect Microbiol. 2025 Mar 10;15:1555171. doi: 10.3389/fcimb.2025.1555171 (PMC11931013; doi:10.3389/fcimb.2025.1555171)
Supplement: Supplementary file 1 [file DataSheet1.pdf]

Supplementary Information

Supplementary Figures

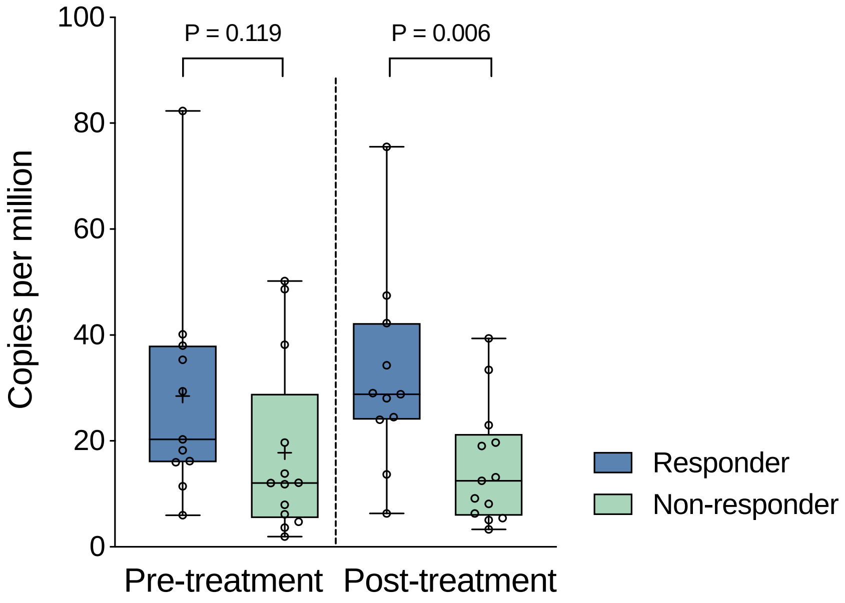

Supplementary Figure 1. Comparison of microbial beta-glucuronidase levels (EC 3.2.1.31) by treatment response before and after treatment. Microbial beta-glucuronidase levels were analyzed based on treatment response, both before and after treatment. The box plot display the median, 25th to 75th percentiles, with whiskers extending from min to max. Statistical significance between groups was calculated using the Mann-Whitney U test.

Supplementary Tables

Supplementary Table 1. Metadata, analysis groupings, and complete taxonomic and functional profiles for all samples. Data provided in MS Excel format.

Supplementary Table 2. Complete differential taxonomic results from LEfSe and Random Forest analyses, including all comparisons and statistics. Data provided in MS Excel format.

Supplementary Table 3. Complete differential functionla results from LEfSe and Random Forest analyses, including all comparisons and statistics. Data provided in MS Excel format.
